# Impact of Medical and Surgical Treatment of Endometriosis on the Cure of Endometriosis and Pain

**DOI:** 10.1155/2014/264653

**Published:** 2014-12-15

**Authors:** Liselotte Mettler, R. Ruprai, Ibrahim Alkatout

**Affiliations:** Department of Obstetrics and Gynecology, University Clinics of Schleswig-Holstein, Campus Kiel, Arnold-Heller-Straße 3/24, 24105 Kiel, Germany

## Abstract

This endometriosis study evaluates three different treatment strategies (hormonal medication, surgical, or combined treatment) and discusses the influence of endometriosis on the cure of this disease and pain relief. Four hundred and fifty patients with genital endometriosis, aged 18–44 years, were randomly distributed to three treatment groups at the first laparoscopy. They were reevaluated at a second-look laparoscopy (D 426/10), one to two months after the three-month hormonal therapy for groups 1 and 3 and five to six months later for group 2 (surgical treatment alone). Outcome data focussed on the recurrence of symptoms and pain. The three treatment options independent of the initial endoscopic endometriosis classification (EEC) stage including deep infiltrating endometriosis (DIE) achieved an overall cure rate of 50% or higher. The highest cure rate of 60% was achieved by the combined treatment, 55% by the exclusively hormonal therapy, and 50% by the exclusively surgical treatment. An overall pregnancy rate between 55% and 65% was achieved with no significant difference in relation to the therapeutical option.

## 1. Introduction

Pelvic endometriosis remains a late diagnosed complex and mysterious disease that still gives rise to continuous research but can currently only be treated in up to 70% of cases with 3 treatment options.

*Medical Treatment.* In the past, the main strategy was the induction of a pseudopregnancy and the application of gestagens and later danazol and GnRH analogues [1]. Up to now, this theory “The production of pseudopregnancy” has been regarded as the “gold standard,” but it is now supplemented by a simple progesterone (dienogest, 2 mg per day) treatment or a GnRh analogue treatment with add-back therapy [2]. To prevent side effects of the GnRH agonist, such as bone demineralization, vasomotor symptoms, and mood swings, a serum estradiol concentration of approximately 60 pg/mL is required [1, 3–5]. Every medical treatment today is well tolerable but should only be used as long as necessary. In case it is used as long-time treatment, it should reduce the number of surgical interventions and improve the quality of life. 

*Inhibition of Mediators.* Research work has focussed on inhibiting the interaction of various mediators which maintain the illness by way of inflammatory processes, vascularisation, and cell proliferation. Specific aromatase inhibitors (such as Letrozole, Anastrozole, or Exemestane) or selective COX-2 inhibitors (e.g., Celecoxib, Rofecoxib) are of great interest and have been studied in clinical trials [6–8]. There is no proven evidence that one medical therapy is superior to another in the treatment of the clinical symptoms of endometriosis or infertility.

**Figure 1:**
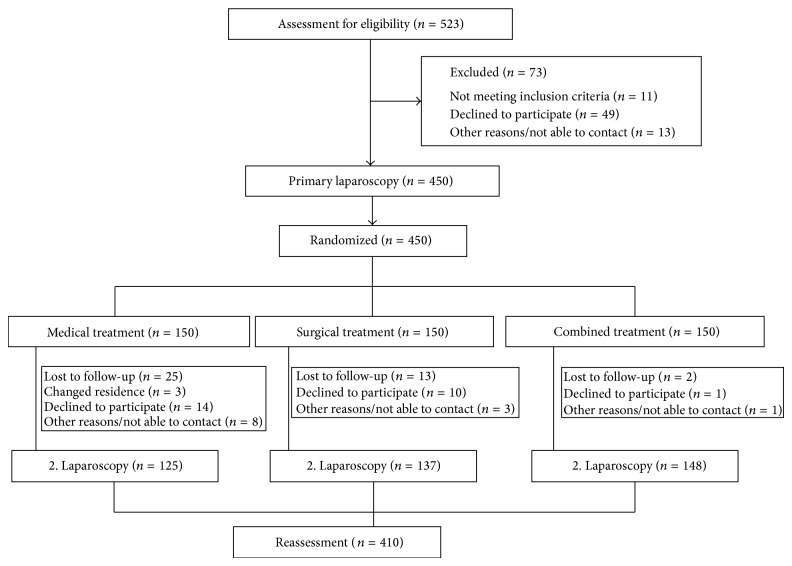
Trial profile differentiating medical, surgical, and combined treatment of endometriosis (with permission of Alkatout et al. [17]).

*Surgical Treatment.* As endometriosis is a progressive disease, which can cause the anatomic destruction of the reproductive organs, surgical therapy plays an important role. Laparoscopy provides the only possibility of ascertaining the expected diagnosis of endometriosis. Endometriosis has a varying phenotype and can appear as raised flame-like patches, whitish opacifications, yellow-brown discolorations, translucent blebs, or reddish irregularly shaped spots (Figure 1) [9, 10]. In advanced stages, pain and sterility are predominantly caused by organ damage, fibrosis, and adhesions, thus constituting a clear indication for surgical intervention. Early laparoscopy can prevent any delays in diagnosis of the disease or symptom progression. The importance of laparoscopy with biopsy and/or resection is reinforced as visual diagnosis alone can often lead to a misdiagnosis [11, 12]. Risk factors and disadvantages of laparoscopy include damage of organs adjacent to the affected areas and postoperative complications, such as adhesion formation or infection [13–17]. Symptom relief is achieved in most patients after successful ablation/resection of endometriosis and adhesiolysis. Nevertheless, the recurrence rate is as high as 40% after a 10-year follow-up [16, 18–20].

*Combined Treatment.* The combined treatment involves diagnostic laparoscopy, removing all visible endometriosis foci as far as possible, a 3- to 6-month endocrine therapy, and a subsequent second-look laparoscopy with resection of residual foci, adhesiolysis, and reconstruction of organs [8, 19–23]. Despite maximal efforts, the therapy of first choice in the management of endometriosis is still unclear [14, 24].

## 2. Material and Methods

In the following we focus on current treatment possibilities, pain, fertility, and the obstetrical outcome in endometriosis patients.

In a recent study, 450 endometriosis patients underwent one of three different therapeutic strategies (medication, surgical, or combined treatment) at the Kiel University Department of Obstetrics and Gynecology. The evaluation aims at determining the most successful of the available endometriosis therapies.

### 2.1. Patients

Informed consent forms were completed by all patients. This study, which included operation, medical treatment, and a selected second-look operation, was approved by the Ethical Committee of the Christian-Albrechts-University Kiel, Germany (D 426/10). Each patient signed an informed consent form for the use of his specimen and clinical data.

The study comprised 450 symptomatic endometriosis patients (18–44 years of age) for whom two consecutive laparoscopic interventions were to be assessed. There were pain and/or infertility patients. 410 patients from the original collective returned for a second-look laparoscopy (Figure 1).

**Figure 2:**
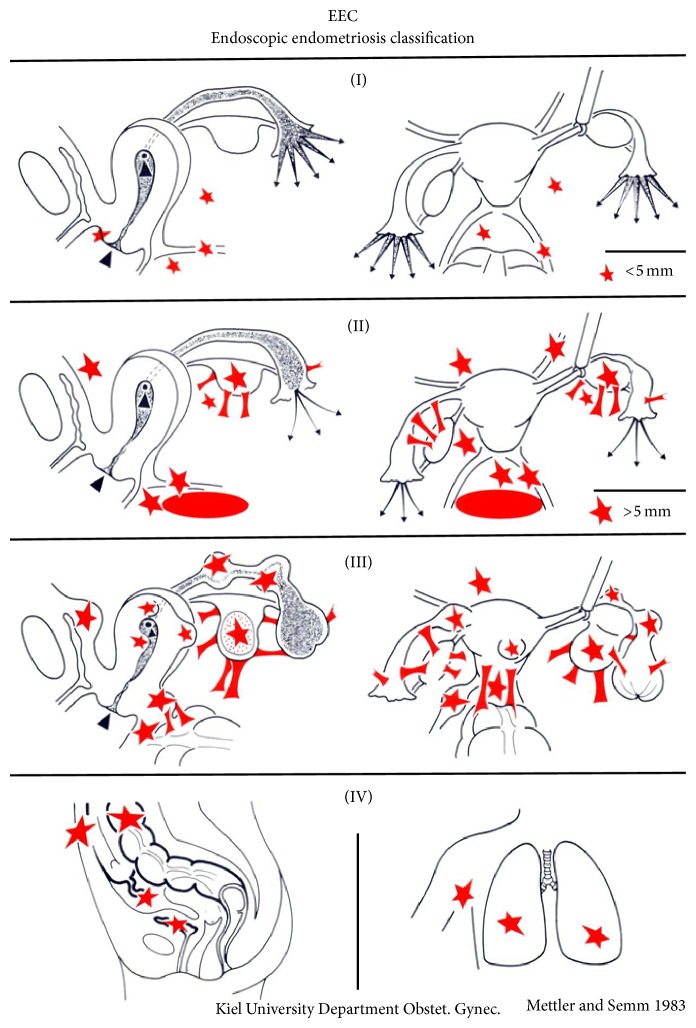
The EEC system used to classify endometriotic lesions. In contrast to the rASRM classification, the EEC classification includes extragenital endometriosis and is divided into four stages.

Endometriosis was diagnosed or confirmed by laparoscopy and rated according to the endoscopic endometriosis classification (EEC) introduced by Mettler (Figure 2) [25] which compares completely to the r-AFS classification. It was used as it is very easy and purely morphologically straightforward.

### 2.2. Exclusion Criteria

Previous surgery or hormone therapy for endometriosis was exclusion criterion, as was deep infiltrating endometriosis with bladder or rectum excision. The treatment of deep infiltrating endometriosis with big lesions affecting bowel and/or urinary tract, favorably diagnosed before surgery, was performed via extensive laparoscopic resection.

**Figure 3:**
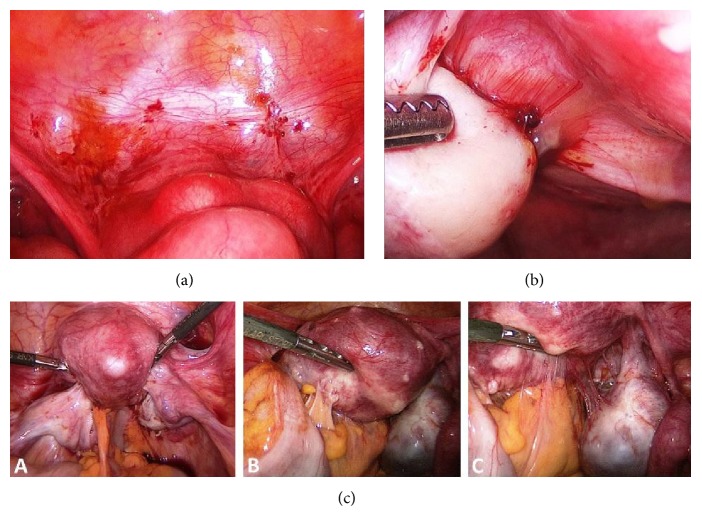
Endoscopic image of endometriosis EEC stage I (a), EEC stage II (b), and EEC stage III ((c): (A)–(C)).

Figure 3 differentiates stages I, II, and III in the laparoscopic appearance.

### 2.3. Tissue Samples

Samples of ectopic endometrium (*n* = 450) were obtained from patients undergoing diagnostic hysteroscopy and laparoscopy for the treatment of endometrioma.

The patients ranged in age from 18 to 44 years and received no hormonal treatment prior to surgery. Cryostat sections were prepared and stained with hematoxylin-eosin. Histopathological assessment confirmed the site of origin, that is, proliferative endometrium or endometrioma cyst wall, respectively.

### 2.4. Interventions

The 450 patients were randomly distributed to the following three treatment groups, 150 per group. Of the original 450 patients, 410 returned for the second-look pelviscopy and their findings were assessed.

Group 1 (*n* = 125) underwent hormonal treatment after diagnostic laparoscopy with 3.75 mg of leuprorelin acetate depot which was injected subcutaneously in monthly intervals over 3 months. Leuprorelin acetate depot is a GnRH agonist and is commercially available in Germany as Enantone Gyn Depot.

Group 2 (*n* = 137) underwent surgical laparoscopy without any subsequent medical treatment. Endometriosis foci were totally excised, adhesions were removed, and the normal anatomy of the reproductive organs was restored. Ureter and superficial bowel lesions were removed. For infertility patients, tubal patency was checked and chromoperturbation was performed at the second-look laparoscopy. Patients with deep infiltrating endometriosis with bladder or rectum resection were not included in the study.

Group 3 (*n* = 148) underwent the same hormonal therapy as group 1 over the same time period after surgical laparoscopy. The combined or three-step therapy comprised diagnostic laparoscopy, removal of all visible endometriosis foci, a 3-month endocrine therapy with GnRH agonists (e.g., 3.75 mg of leuprorelin acetate depot), and a subsequent second-look laparoscopy 1-2 months after conclusion of the hormonal therapy with resection of residual foci and reconstructive surgery of organs.

The same team of physicians performed the primary and secondary intervention as well as the primary and secondary endometriosis staging according to the EEC [25, 26]. For groups 1 and 3, a second-look laparoscopy was performed 1-2 months after hormonal therapy and, for group 2, 5 to 6 months after surgical endometriosis treatment. After the second-look laparoscopy, patients were monitored over a period of 2 years and completed an extensive questionnaire to determine their recurrence of symptoms, new endometriotic lesions determined laparoscopically, and confirmed pregnancy rates. Also patients in group 2 were reevaluated with a second-look laparoscopy as endometriosis may reappear.

### 2.5. Main Outcome Measures

The central issue for this study was, Which endometriosis therapy is currently the most successful technique? The success of each therapeutic strategy was assessed—independent of the original EEC stage—according to the following criteria after the second-look laparoscopy:

1. a response rate to EEC stages 0 and I of at least 75%,
2. the lowest recurrence rate,
3. the highest pregnancy rate.

Within the framework of this study, the endometriosis therapy that fulfilled all of the criteria or at least two of them was regarded as the most successful therapy.

### 2.6. Statistical Evaluation and IRB Approval

Our results were statistically evaluated with the chi-squared test and analysed with a significance level of *P* < 0.05 and a confidence interval of 95%. Institutional review board approval was obtained at the beginning of the study.

## 3. Results for Extent of Endometriosis, Fertility, and Pain

Results in the 3 treatment groups were analyzed to assess the new endometriosis staging or EEC downstaging. There was no significant difference between the groups insofar as distribution of EEC stages before treatment. After the individual treatment, the distribution of EEC stages indicated a significant difference between the 3 groups (*P* = 0.01). The shift in the CI is indicative of a higher rate of cure in patients in group 3 compared with groups 1 and 2. This was most remarkable for stage EEC 0 (Table 1). The definition of cure rate is that laparoscopically there were no more endometriotic lesions visible for stage EEC 0.

At the onset of the study, in the 125 patients in group 1 (hormone therapy), disease stage was EEC I in 40%, ECC II in 38%, and EEC III in 22%. After hormone therapy and independent of the previous EEC stage, disease stage was EEC I in 32% of patients, EEC II in 13%, and EEC III in 5%. In 50% of the patients, second-look laparoscopy showed no signs of endometriosis (EEC 0). In these patients, the disease seemed to be cured (cure rate = 50%). In the 137 patients in group 2 (surgical treatment), disease stage was EEC I in 50%, EEC II in 32%, and EEC III in 18%. At second-look laparoscopy, the disease could be downstaged to EEC I in 13% of patients, EEC II in 23%, and EEC III in 9%.

The cure rate for the exclusively surgically treated group was 55% (EEC 0). In the 148 patients in group 3 (combined treatment), disease stage was EEC I in 53%, EEC II in 24%, and EEC III in 23%. After combined surgical and hormone therapy, disease stage was EEC I in 18% of the patients, EEC II in 17%, and EEC III in 5%. With combined treatment, the cure rate was 60% (EEC 0).

The 3 treatment options achieved, independent of the initial EEC stage, an overall cure rate of ≥50%. With combined treatment, the cure rate was 60%, with exclusively hormonal therapy was 55%, and with exclusively surgical treatment was 50%. Within the framework of the study, cure was defined as a reduction in disease stage to EEC 0. This new endometriosis downstaging was confirmed at second-look laparoscopy. The best total cure rate was achieved with combined treatment (Table 1).

In a second step, we differentiated light, intermediate, and advanced endometriosis and evaluated therapeutic strategies. An improvement of at least 75% to EEC stage 0 or stage 1 was defined as highly efficient. These conditions were met with the exclusively hormonal therapy, with a rate of 82% (50% EEC 0 and 32% EEC I), and with the combined treatment (3-step therapy), with a response rate of 78% (60% EEC 0 and 18% EEC I) (Table 1).

Because endometriosis generally causes recurrent pain, we asked our study patients to complete an extensive questionnaire and report recurrent symptoms, before and at 1 year after the end of all therapeutic activities as a second outcome measure [27].

Results in the 3 treatment groups were analyzed to assess the treatment effect considering the recurrence of symptoms of endometriosis. There was no significant difference between the groups insofar as distribution of symptoms before treatment (*P* = 0.61 for dysmenorrhea, *P* = 0.59 for dyspareunia, and *P* = 0.54 for abdominal pain).

After the individual treatment, the distribution of recurrence of symptoms highlights a general reduction in symptoms, with the greatest benefit observed in the combined treatment group. There was a difference, statistically not significant, for dysmenorrhea between the therapeutic groups (*P* = 0.05) after treatment. The 95% CI demonstrated a remarkable difference in the treatment effect in all 3 groups. Nevertheless, the treatment effect was strongest in group 3, followed by group 2. Insofar as dyspareunia, a significant difference was noted between the 3 treatment groups (*P* = 0.007). The CIs demonstrated the biggest treatment effect in group 3, followed by group 2. Abdominal pain could not be reduced significantly (*P* = 0.284). Nevertheless, the CIs showed the biggest effect in group 3, followed by group 1. In group 1 (hormone therapy), at the onset of the study, 60% of the 125 patients had dysmenorrhea, 56% had dyspareunia, and 48% had abdominal pain. The group that received exclusively hormonal therapy had the highest recurrence rates: dysmenorrhea in 28% of the patients, abdominal pain in 26%, and dyspareunia in 22%. In group 2 (surgical treatment), 57% of the 137 patients had dysmenorrhea, 50% had dyspareunia, and 42% had abdominal pain. After follow-up, 20% of the women in this group reported dysmenorrhea, 15% reported dyspareunia, and 24% reported abdominal pain.

**Figure 4:**
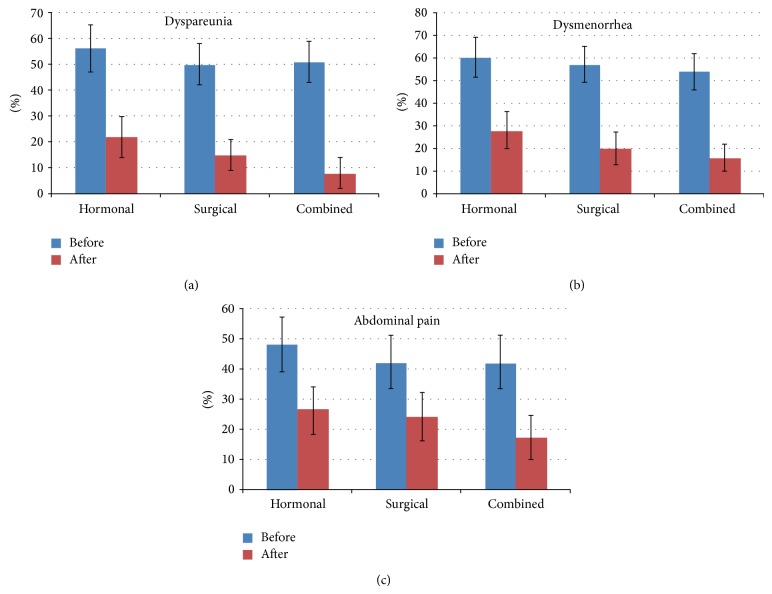
Comparison of recurrence rates of symptoms including dyspareunia (a), dysmenorrhea (b), and abdominal pain (c) for each of the 3 treatment groups before 1 year after treatment. Therapeutic benefit is supported by the marked confidence intervals.

In group 3 (combined treatment), 54% of the 148 women had dysmenorrhea, 51% had dyspareunia, and 42% had abdominal pain. Patients in the combined treatment group achieved the lowest general recurrence rate and the lowest recurrence rate per symptom: 16% of the patients reported dysmenorrhea, 8% reported dyspareunia, and 17% reported abdominal pain at 1-year follow-up (Figure 4). In comparison with groups 1 and 2, group 3 had significantly better results after treatment (*P* < 0.001).

The third outcome measure was the pregnancy rate. We determined an overall pregnancy rate over 2 years of 55% to 65% in the 3 treatment groups, independent of ECC stage (Table 2). The pregnancy rate after the exclusively surgical restoration was 55%, after combined treatment was 60%, and after exclusively hormonal therapy was 65%.

There was no statistical significance between these results.

Of these 245 pregnancies, 41 (17%) were not carried to term (6 ectopic pregnancies and 35 abortions). However, 205 children, including 1 set of twins, were born. There was no statistically significant difference between the 3 therapeutic strategies insofar as the pregnancies and their course.

## 4. Discussion

The presented clinical studies comparing medical, surgical, and combined therapy and the assessment of how endometriosis can affect pregnancy and deliveries show the current needs for the treatment of endometriosis and point out some advice for future therapeutic modalities.

In the presented study, 450 endometriosis patients, aged 18–44 years, were randomly assigned to one of the three different therapeutic strategies (medical, surgical, or combined treatment) at the Kiel University Department of Obstetrics and Gynecology, Germany. The success of each therapeutic strategy was assessed—independent of the original EEC stage [25]—according to the following criteria:

- the therapy after which the patients achieved the highest cure rate (EEC stage 0),
- a response rate to EEC stages I and 0 of 75% or higher,
- the lowest recurrence rate,
- the highest pregnancy rate.

Within the framework of this study, the endometriosis therapy that fulfilled the majority of the criteria, or at least two of them, was regarded as the most successful therapy. The three treatment options reached an overall cure rate of 50% or higher. There was no statistically significant difference (*P* > 0.05), but with a cure rate of 60% the combined therapy ranks first. The combined (three-step) and the exclusively hormonal therapy managed to surpass the 75% response rate with 78% and 82%, respectively. Nevertheless, the combined treatment reached the lowest recurrence rate per symptom at a statistically significant level. No statistically significant difference was recorded for the pregnancy rate which ranged between 55% and 65%, independent of the therapeutic strategy. As an overall result, we have been able to confirm the high efficacy of the combined endometriosis therapy in this study.

Medical therapy can be applied prior to surgery to decrease the size of endometriotic implants and the extent of the operation [28]. However, so far there is no clear evidence that perioperative hormonal treatment decreases the extent of operation necessary to remove endometriotic implants, delays or prevents recurrence, or increases pregnancy rates. In contrast, several trials were able to report an increased duration of pain relief and delayed recurrence rates using postoperative medical therapy [10, 28, 29]. Schweppe concluded that, in all cases of active endometriosis, pelviscopic treatment alone is not sufficient [30]. Schindler et al. demonstrated that the primary surgical intervention reduced the total r-AFS (revised American Fertility Society) score by 34%, whereas the combined therapy brought about a reduction of 66% [31].

Thus the combined hormonal and surgical treatment of endometriosis gave best results in stopping the progressing of the disease, as already found by our group [32].

Our study showed only a weak and statistically nonsignificant difference between the combined treatment (decrease in EEC stage by 60%) and the solitary surgical treatment (decrease in EEC stage by 55%).

Regidor found a significant improvement of the r-AFS score after treatment with triptorelin (GnRH analogue). Sixty-three percent of these patients were no longer diagnosed with endometriosis, 30% presented with stage I residual endometriosis according to the AFS classification, and only 7% had stage II endometriosis [33]. It could be demonstrated that after administration of buserelin (GnRH analogue) the average AFS score went down from 17.4 ± 12.9 before therapy to 7.2 ± 8.2 after a 6-month therapy [31, 34]. Although up to 90% of patients experience some symptom relief with medical therapy, medical treatment alone neither enhances fertility, diminishes pelvic mass, nor removes adhesions [1, 10].

Similar to Schweppe [30] and Römer and Schwesinger [35], we also determined a significantly lower recurrence rate after application of the three-step therapy. Römer and Schwesinger reported that retrospective analyses 12–48 months after endometriosis therapy presented a recurrence rate for hormonal and surgical treatment (three-step concept) of only 16.7%, whereas the recurrence rate for exclusively surgically treated patients was 47%.

Regidor et al. showed in a long-term follow-up study that 70 of 112 patients (62.5%) again reported ailments and that the recurrence-free interval amounted to an average of 11 months after finishing the three-step therapy (with the GnRH analogue leuprorelin acetate) [36]. In another long-term follow-up study, Schindler et al. established recurrent endometriosis in 62 of 112 patients (55%) after a combined surgical-hormonal therapy [1, 37]. Our recurrence rate (41%) was lower than Regidor's rate (62.5%) and Schindler's rate (55%) for the combined therapy. Zupi et al. were able to show that patients treated with GnRH agonists had a significantly higher rate of symptom reduction (pelvic pain, dysmenorrhea, and dyspareunia) than women treated with continuous estrogen-progestin oral contraceptives. Quality of life was increased by extending the GnRH treatment to include add-back therapy [3]. Other investigations comparing oral contraceptives to GnRH agonists found an equal reduction of pain [38]. Abbott et al. 2004 and Sutton et al. 1994 performed a second-look laparoscopy 6–12 months after the primary operation and found that 29–45% of the patients had disease progression and 22–29% disease regression, and in 33–42% the disease remained static [18, 39].

Endometriosis can reduce the fecundability rate without completely preventing conception. Impaired fertility might be due to anatomic variations after adhesion formation and endometriomas [40]. An enhancement of fertility rates through ovulation suppression has not yet been proven [1]. In our study, after the combined therapy, we had 89 (60%) pregnancies in 148 patients and 13 abortions and 3 extrauterine pregnancies. Sixteen (18%) of the 89 pregnancies did not lead to a live birth (13 abortions and 3 extrauterine pregnancies). Regidor reported 55 (60%) pregnancies for 91 patients for the same therapeutic strategy [36]. Nineteen (34.5%) of the 55 pregnancies were not carried to term (5 extrauterine pregnancies, 14 abortions). Our pregnancy rate was comparable to Regidor's rate, but our abortion rate was significantly lower than his. After the exclusively surgical treatment, we registered a pregnancy rate of 55%. In comparison, Marcoux et al. presented a pregnancy rate of 29% [41].

All researches focussing on macroscopic or microscopic markers as well as biochemical criteria for assessing the degree of activity of endometriosis are not convincing. Essential factors for deciding the optimal endometriosis therapy are clinical symptoms, the patient's age, localization, severity, duration of the disease, recurrence rate, and activity [42, 43]. Active endometriosis foci are characterized by hypervascularisation, oedema, and infiltration of inflammatory cells [44]. It still needs to be determined how endometriosis activity can best be characterized using macroscopic, microscopic, and biochemical criteria [15]. Laparoscopy currently constitutes one of the most accurate methods of diagnosing endometriosis. As we are continuously engaged to better diagnose and treat our endometriosis patients, the reevaluation of our study of 2013 [32] made us again aware of the limited therapeutic armamentarium available to successfully treat this enigmatic benign disease. We can only hope for molecular genetics and proteomics to make research possibilities soon available for the diagnosis and treatment of endometriosis.

## 5. Conclusions

Since the identification of endometriosis as a progressive estrogen-related disease, various substances have been used to suppress ovarian steroid biosynthesis. Currently all modern therapeutic strategies aim at ovarian downregulation with GnRH agonists or gestagens. In most cases, therapeutic approaches take into consideration not only medical but also laparoscopic and, if required, laparotomic surgical treatment of endometriosis and the combined therapy. The three-step therapy comprises surgical laparoscopy with removal of all visible endometriosis foci, a 3- to 6-month endocrine therapy, and a subsequent second-look laparoscopy with resection of residual foci, adhesiolysis, and reconstructive surgery of the organs [45]. Within the framework of the present study, combined treatment was the most successful treatment for endometriosis. Comparison of the 3 different therapeutic strategies implicates a higher benefit for combined treatment insofar as downstaging and reduction in symptoms (disease-free period) and pregnancy rates.

## Figures and Tables

**Table 1:** Distribution of patients to EEC stages before (*P* = 0.105) and after therapy (*P* = 0.010).

Therapy methods	EEC stage							
	EEC 0	CI	EEC I	CI	EEC II	CI	EEC III	CI
Group 1, hormonal (*n* = 125)								
Before therapy	0	—	50 (40%)	31.3–49.1	47 (38%)	29.1–46.7	28 (22%)	15.4–30.7
After therapy	62 (50%)	40.5–58.7	40 (32%)	23.9–40.9	16 (13%)	7.4–20.0	7 (5%)	2.3–11.2
Group 2, surgical (*n* = 137)								
Before therapy	0	—	69 (50%)	41.7–59.0	44 (32%)	24.4–40.6	24 (18%)	11.5–24.9
After therapy	75 (55%)	46.0–63.3	20 (13%)	9.2–21.6	30 (23%)	15.3–29.8	12 (9%)	4.6–14.8
Group 3, combined (*n* = 148)								
Before therapy	0	—	79 (53%)	45.0–61.1	36 (24%)	17.7–32.1	33 (23%)	15.9–29.9
After therapy	89 (60%)	51.3–68.1	26 (18%)	11.8–24.7	25 (17%)	11.2–23.9	8 (5%)	2.4–10.4

CI: confidence interval.

**Table 2:** Comparison of recurrence rates for the three therapy methods before and after one year.

Therapy methods	Recurrent symptoms (in %)/CI					
	Dysmenorrhea		Dyspareunia		Abdominal pain	
	Before (*P* = 0.613)	After (*P* = 0.053)	Before (*P* = 0.0593)	After (*P* = 0.007)	Before (*P* = 0.0540)	After (*P* = 0.284)
Group 1, hormonal (*n* = 125)	75 (60%)/50.9–68.7	35 (28%)/20.3–36.7	70 (56%)/46.8–64.9	28 (22%)/15.4–30.7	60 (48%)/40.0–57.1	33 (26%)/18.9–35.0
Group 2, surgical (*n* = 137)	78 (57%)/48.2–65.4	27 (20%)/13.4–27.3	69 (50%)/41.7–59.0	21 (15%)/9.7–22.5	58 (42%)/33.9–51.1	33 (24%)/17.2–32.1
Group 3, combined (*n* = 148)	80 (54%)/45.7–62.3	24 (16%)/10.7–23.2	75 (51%)/42.3–59.0	12 (8%)/4.2–13.7	62 (42%)/33.8–50.3	25 (17%)/11.2–23.9

CI: confidence interval.
